# Metacognition in human decision-making: confidence and error monitoring

**DOI:** 10.1098/rstb.2011.0416

**Published:** 2012-05-19

**Authors:** Nick Yeung, Christopher Summerfield

**Affiliations:** Department of Experimental Psychology, University of Oxford, South Parks Road, Oxford OX1 3UD, UK

**Keywords:** decision-making, confidence, metacognition, error monitoring

## Abstract

People are capable of robust evaluations of their decisions: they are often aware of their mistakes even without explicit feedback, and report levels of confidence in their decisions that correlate with objective performance. These metacognitive abilities help people to avoid making the same mistakes twice, and to avoid overcommitting time or resources to decisions that are based on unreliable evidence. In this review, we consider progress in characterizing the neural and mechanistic basis of these related aspects of metacognition—confidence judgements and error monitoring—and identify crucial points of convergence between methods and theories in the two fields. This convergence suggests that common principles govern metacognitive judgements of confidence and accuracy; in particular, a shared reliance on post-decisional processing within the systems responsible for the initial decision. However, research in both fields has focused rather narrowly on simple, discrete decisions—reflecting the correspondingly restricted focus of current models of the decision process itself—raising doubts about the degree to which discovered principles will scale up to explain metacognitive evaluation of real-world decisions and actions that are fluid, temporally extended, and embedded in the broader context of evolving behavioural goals.

## 1. Introduction

### (a) Metacognition in perceptual choice

Imagine yourself cycling along a narrow, winding country lane on a summer's day. Successfully negotiating each twist and turn in the road requires the interpretation of a variety of subtle sensory cues and their conversion into appropriate motor commands. For example, incoming visual signals that reveal the curvature of the road, or motion parallax signals arising from the trees beyond, allows you to gently adjust the handlebars towards the left or right to steer the bicycle smoothly round each bend.

Over past decades, psychologists and neuroscientists have devoted substantial effort to understand the neural and computational mechanisms by which actions are selected on the basis of a stream of incoming sensory information [1]. Because this sort of sensorimotor control often requires an observer to commit to one discrete action from among several possible candidates, this literature has been informed by computational models that offer a formal account of how categorical decisions are made. These models, described in more detail below, have provided an excellent account of the decisions and decision latencies exhibited by observers selecting actions on the basis of ambiguous sensory information [2–5].

However, now imagine that as you are cycling, the sky darkens and it begins to rain—visibility is reduced, and the road becomes slippery and wet. The approach to each bend still requires a deft angling of handlebars, but now you are less certain about whether each chosen action is optimized to steer the bicycle in a fashion that appropriately meets the gradient of the curve and the camber of the road. Action selection proceeds as before, but you are unsure whether the actions chosen are the most appropriate ones. In fact, you might even experience an immediate sense that you have just made a poor choice. In short, you are less *confident* about your decisions.

Curiously, despite universal agreement that an accompanying sense of confidence is a subjectively salient property of almost all our decisions, there is currently little consensus about how we might incorporate decision confidence into formal models of choice behaviour or explore its biological substrates. Fundamental questions remain unanswered. For example, is the information that gives rise to the ‘second-order’ estimate of confidence in a choice identical to that determining the ‘first-order’ choice itself? Why are we generally more sure that we are correct than that we have made an error, even for difficult choices? Why do we sometimes appear to ‘change our mind’ after a motor programme has been initiated? Are these changes of mind necessarily accompanied by awareness that the initial choice was incorrect?

In what follows, we review the literature that has posed these and related questions about decision confidence in perceptual choice tasks. Subsequently, we highlight links between this work and a literature that has considered how people monitor whether they have made an error under conditions of uncertainty or conflict. Finally, we propose some potentially fruitful avenues for research that draw upon common themes in these two literatures, building on their shared strengths and addressing their shared limitations.

### (b) Formal models of perceptual choice

**Figure 1.**
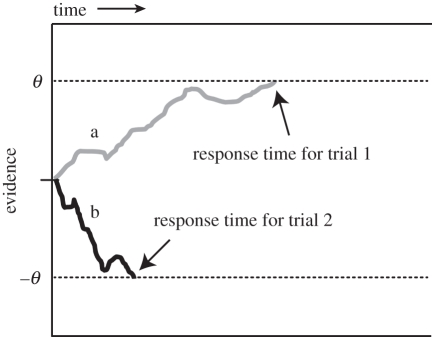
The drift-diffusion model. Accumulating evidence (the decision variable, *y*-axis) over time (*x*-axis) is shown for two illustrative trials (marked a and b, grey and black lines), one on which the choice *θ* is made and the other on which the choice –*θ* is made. A decision is triggered when evidence reaches *θ* or –*θ*. Grey line, trial 1; black line, trial 2.

Even under good viewing conditions, visual information is corrupted by multiple sources of noise and uncertainty, arising both in the external world and in the dynamics of neural processing. One sensible way to increase the signal-to-noise ratio of visual information is to sample the external world repeatedly and integrate this information over time, making a decision only when the information is considered to be of sufficient quality [5]. This idea forms the basis of a class of model in which binary choices are described via an accumulation-to-bound mechanism, with successive samples of information totted up until they reach a criterial level or ‘bound’, upon which a commitment to action is made (figure 1).

Different versions of this model make divergent assumptions about exactly what quantity is integrated *en route* to a decision—i.e. about precisely how the ‘decision variable’ (DV) is composed. To simplify matters, in this review, we focus on one variant of this model—the ‘drift-diffusion’ model, or DDM—in which the DV corresponds to the relative likelihood of the two choices being correct, given the stimulus [3]. In the DDM, the DV on sample *v_t_* is updated on each sample *t* with an increment composed of two quantities: *δ*, a linear drift term that encodes the rate of evidence accumulation, and *cW*, Gaussian noise with a mean of zero and a variance of *c*^2^:1.1
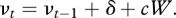


Decisions are made when the DV exceeds a fixed deviation from zero, *θ*, such that during evidence accumulation:1.2
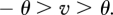


This simple model has much to recommend it both as a normative and descriptive account of categorical choice. The DDM has been widely and very successfully applied to decision-making in a range of cognitive domains—from low-level perceptual decisions to retrieval of facts from semantic memory, to economic decision-making under uncertainty [6–8]—and accounts neatly for several empirical characteristics of reaction times (RTs) observed for binary choices in these tasks. Firstly, the natural geometry of the accumulation-to-bound process predicts the observed rightward skew in RT distributions. Secondly, varying the bound offers an elegant account of the economy of speed and accuracy that characterizes mental chronometry tasks. Thirdly, by allowing drift rate and origin of accumulation to vary across trials, the model accounts for the relative RT of correct and error trials under conditions where speed or accuracy are emphasized [8]. Moreover, the model implements a sequential probability ratio procedure that optimizes speed for a given error rate, and thus takes its place among a family of statistically optimal descriptions of the choice process [2].

## 2. Decision confidence

However, as illustrated above, we not only make decisions, but also concurrently evaluate the likelihood that those decisions will result in favourable or unfavourable outcomes. How, then, can we incorporate decision confidence into the formal framework offered by the DDM and other quantitative models of perceptual choice? In what follows, we review recent empirical and modelling work that has attempted to extend this formal framework to account for metacognitive judgements. Much of this debate has hinged on a simple question: can confidence be read out directly from the DV at the time of choice (decisional locus model), or do confidence judgements depend on new information arriving beyond the decision point (post-decisional locus models)? [9].

As an aside, we note that this question only arises if one is concerned with modelling the dynamics of the decision process, such as with the DDM or related evidence-accumulation models. Other decision-theoretic models, such as signal detection theory (SDT), have given detailed treatment of how to measure observers' metacognitive sensitivity, specifically, their ability to distinguish their own correct and incorrect judgements [10–12]. However, because SDT is a static model of the decision process in which the temporal dynamics of evidence accumulation are ignored, primary (‘type I’) and metacognitive (‘type II’) judgements must necessarily be based on the same evidence (albeit potentially corrupted by different sources of noise [12]), and thus these models are by definition decision locus models.

### (a) Decisional locus models

The very earliest investigations of decision confidence revealed that, perhaps unsurprisingly, we are more certain about our perceptual choices when sensory inputs are stronger [13], and when we are given longer to sample sensory information [14]. It thus follows that confidence should reflect both the quality of the evidence (represented by the drift rate, *δ*) and the quantity of evidence at the choice point (represented by the absolute value of the decision bound, |*θ*|). However, it is also clear that neither of these quantities alone is sufficient to describe an observer's confidence in their choices. Any diffusion-to-bound model assuming that confidence is directly indexed by the state of evidence at the time of choice inevitably predicts that all choices will be made with precisely the same confidence (corresponding to the evidence level required to reach the bound). Similarly, any model proposing that confidence reflects evidence quality implicitly assumes that observers have direct access to this quantity—which, if they had, would obviate the need for a sequential sampling approach in the first place (see Pleskac & Busemeyer [15] for an excellent recent review).

Various accounts dating back over 100 years have therefore proposed that confidence reflects some interaction between the quantity and quality of evidence—for example, that under the DDM, confidence scales with the product of *δ* and *θ* [16,17]. This account has attractive properties—for example, it predicts that when the observer has the option of terminating evidence accumulation with a choice at any point, RTs are faster for high-confidence trials [18]. Moreover, this view enjoys support from two prominent neurophysiological studies detailed in §2*b*.

### (b) Neural substrates of decision confidence

Two recent single-cell recording studies, one involving rats [19] and the other monkeys [20], claim to have identified neurons encoding subjective decision confidence. Kiani *et al*. [20] recorded from neurons in the lateral intraparietal cortex (LIP) of macaques discriminating the direction of motion in ambiguous moving-dot arrays [21] with a saccade to one of two targets placed either side of the motion stimulus. This experimental approach has previously been used to demonstrate that LIP neurons whose receptive field overlaps with the chosen target display a characteristic acceleration of spiking activity while the monkey views the moving-dot stimulus, whereas those overlapping with the non-chosen target show a relative dampening of activity [22,23]. This build-up scales with signal strength, and terminates when an action (saccadic eye movement) is selected, prompting the view that LIP firing rates encode the level of evidence available for a choice—in other words, their firing rates form a neural representation of the DV proposed by the DDM [1].

To investigate decision confidence, Kiani *et al.* added a new twist to this protocol. Typically, the monkey is rewarded for correct but not incorrect choices, but here, on a fraction of trials, the monkey was offered a ‘sure bet’ option such that a certain but lower-valued reward could be obtained via a saccade to a third response option. In ethology, it is commonly assumed that an animal choosing the safe option when evidence is scarce must have access to metacognitive information about the likelihood that it will make an error on the main task [24]. The authors found that not only did their monkeys use this option judiciously—responding when the stimulus was weak or ambiguous—but also that LIP firing rates on low-confidence trials were substantially attenuated, falling equidistant between those for the chosen and unchosen targets. The authors interpret these data as showing that confidence is a simple product of quantity and quality of the DV, as proposed by early accounts of decision confidence, obviating the need for a separate metacognitive monitoring process [20].

Kepecs *et al.* [19] employed an odour discrimination paradigm in conjunction with single-cell recordings in the rat orbitofrontal cortex (OFC; see also Kepecs & Mainen [25]). They report an intriguing finding: that OFC firing rates discriminated between correct judgements and errors after the choice but *before* the outcome had been revealed, even when objective difficulty of the choice paradigm was controlled for. Because variance in these responses could not be explained by other factors, such as the reinforcement history over past trials, the authors suggest that these neurons encode confidence estimates associated with the current decision. They show that the activity of these neurons can be explained by a class of model in which two evidence tallies race to the decision bound (rather than a single quantity representing their difference, as in the DDM). Specifically, neuronal responses reflected the difference between the two totals at the time of the decision—making this a decisional locus model, even though the relevant neural activity was sustained through the post-response period—and this activity in turn predicted the likelihood that the choice was correct [19].

At first glance, these two studies offer convincing evidence that neurons in the parietal and orbitofrontal cortices encode the subjective confidence associated with a choice: a graded quantity that can be estimated directly from the evidence on which the original, first-order choice was based. In both cases, the authors are assiduous in attempting to rule out alternative explanations of their findings, such as attention or learned reward contingencies. However, to return momentarily to our discussion of models of categorical choice, two well-replicated behavioural phenomena cast doubt on any model in which subjective confidence directly reflects the evidence accumulated up to the choice point. First, it has consistently been shown that confidence in correct choices is stronger than confidence in incorrect choices, even when choice difficulty is controlled. As previously pointed out [15], this finding cannot be explained by decisional locus models in which confidence is a mere function of diffusion model parameters, because these parameters are invariant across correct and incorrect trials (but see Galvin *et al*. [10] for relevant analysis using SDT). Second, and crucially, subjects occasionally change their mind between the first-order choice and the second-order estimation of decision confidence. At least on these trials, the decisions *must* be influenced by processing that occurs after the first-order choice point. In §2*c*, we consider models that propose such a mechanism.

### (c) Post-decisional locus models and changes of mind

Resulaj *et al*. [26] report a behavioural experiment in which human subjects indicated the direction of a random dot motion stimulus by moving a handle to a leftwards or rightwards target some 20 cm away. This design allowed the researchers to isolate trials on which subjects began to move towards one target but then changed their mind and veered-off towards the other. Careful behavioural analyses demonstrated a number of interesting phenomena. Firstly, changes of mind were not symmetric: subjects switched more often from an incorrect to a correct choice. Secondly, change-of-mind trials tended to occur when, owing to stochasticity in the stimulus display, motion energy began by favouring the initial choice, but subsequently came to favour the alternative, switched-to option. Because the motion stimulus offset once movement began, subjects must have capitalized on the balance of information in the immediate pre-decision period when deciding to change their mind. Notably, although this motion information was available prior to the decision, the switch occurred only once movement initiation began, suggesting that evidence accumulation continued beyond the point at which the initial choice was made.

To account for these and other data, a number of researchers have proposed models in which, in contrast to the classical DDM, evidence accumulation continues even beyond the choice point, with this extra variability in the DV also contributing to estimates of subjective confidence when probed at a later time point [9,15]. Resulaj *et al.* propose that their data can be explained by just this type of model, with changes of mind occurring when latent information in the processing pipeline drives the DV across a second, ‘change-of-mind’ bound. A related account, the two-stage dynamic signal detection (2DSD) model [15], likewise proposes that the diffusion process continues beyond initial choice, with decision confidence reflecting the absolute value of the DV at the post-decision point at which a second-order decision is required. Thus, in contrast to discrete changes of mind that are expressed as overt corrections of an initial response, the 2DSD model allows for continuously varying levels of confidence that provide an explicit judgement about an earlier response.

Behaviourally, these post-decision processing models are able to account for a broad range of findings concerning decision confidence. Firstly, they correctly predict that observers will change their mind more often from incorrect to correct responses than vice versa, because beyond the bound the DV on error trials will tend to regress towards the mean, whereas after correct responses it will continue to grow, driven by the true underlying drift rate. This observation also naturally explains another conundrum associated with decision confidence: that second-order confidence is generally higher for correct trials than incorrect trials. Indeed, reconsidering for a moment the data from Kiani *et al.*, we note that these authors report that neural activity in the delay between stimulus offset and movement execution exerted a separate, independent influence over the decision to choose the ‘sure bet’ option [20]. Thus, although the authors argue that a mechanistic description of decision confidence does not require us to invoke a distinct metacognitive process separate from evidence accumulation, the evidence predicting decision confidence might not be confined to the stimulation period alone. However, one potential caveat to this view is that the activity of LIP neurons is known to drop off sharply once the eye movement is made [22,23]. Thus, it remains to be shown whether variation in the post-decisional LIP signal could contribute to a later confidence judgement, or whether a separate representation of the evolving DV is supporting the observed changes of mind. This caveat aside, these emerging findings suggest that post-decisional processing plays a crucial role in metacognitive judgements, which can lead to changes of mind or support ratings of confidence in an initial decision.

## 3. Error monitoring

People are often aware of their own mistakes; for example, in choice RT tasks when time pressure is applied to induce errors in simple judgements. *Error monitoring* is the metacognitive process by which we are able to detect and signal our errors as soon as a response has been made. This process plays a crucial role in adaptive human behaviour, allowing our actions to be shaped by their outcomes both in the short term, for example, by responding more cautiously to avoid further errors, and in the longer term, through gradual learning of appropriate stimulus–response contingencies.

Whereas the studies described above have typically asked subjects to report their confidence that they made the *correct* choice, studies examining error monitoring have tended to ask subjects the converse question—i.e. to report the likelihood that they made an *error*. Although these judgements seem like two sides of the same coin, methods and assumptions in the two literatures have often been quite different. For example, decision confidence has typically been studied using tasks that remain challenging even when extended processing times are permitted, such as challenging psychophysical discriminations. Under these circumstances, subjects are sometimes sure they responded correctly and sometimes unsure whether they are right or wrong, but rarely certain that that they made a mistake [15]. In contrast, research on error monitoring has mostly been studied using simple but time-pressured tasks in which subjects are usually aware of their errors and very rarely unsure whether their decision was right or wrong. Framed in terms of the DDM, the distinction concerns whether errors and sensitivity to processing noise arise because of low drift rate, *δ*, in the case of perceptual ambiguity, or adoption of a low threshold, *θ*, to engender fast responding. There is nonetheless obvious similarity between metacognitive judgements of confidence and error likelihood, and it is therefore unsurprising that models of error monitoring turn out to complement those more recently developed in perceptual decision-making research.

### (a) Post-decision processing in error monitoring

Rabbitt and co-workers' pioneering work beginning in the 1960s established that error monitoring relies on post-decision processing. Their experiments showed that people can very reliably detect and correct their own errors without requiring explicit feedback [27], but that this ability is impaired when stimulus duration is reduced [28], suggesting its dependence on continued processing of the stimulus after an initial error (which is curtailed when stimuli are very briefly presented). Error monitoring is also impaired when subsequent stimuli appear soon after the initial response [29], and responses to those later stimuli are themselves postponed following errors [30], consistent with the notion that this monitoring involves the same mechanisms as the initial decision.

Summarizing these findings, Rabbitt likened evidence accumulation in decision-making to votes in a committee, in which incorrect decisions are sometimes made on the basis of incomplete information, but ‘as subsequent votes come in, a more accurate consensus will accumulate and the earlier mistake will become apparent’ [28]. Thus, errors are characterized by biphasic evidence accumulation, with initial accumulation in favour of the incorrect response followed by later drift towards the correct decision (as the trial-average drift rate regresses to the true mean). By contrast, continued evaluation following correct responses tends simply to reinforce the original decision. This model is an obvious precursor to more recent accounts of decision confidence [15] and changes of mind [26].

**Figure 2.**
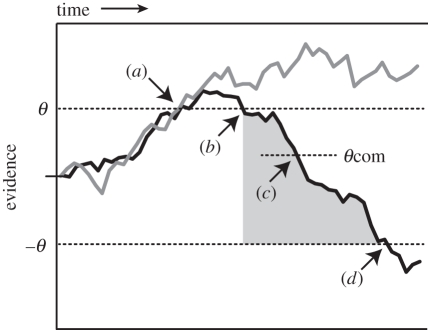
Theories of error detection within the DDM framework. The drift-diffusion process is illustrated schematically for two trials, one in which decision *θ* is the correct response and one trial in which this decision is incorrect. Both decisions occur at the same time point (*a*). Following the correct response (grey line), post-decision processing continues to accumulate in favour of the decision just made. Following errors (black line), the drift rate regresses to its true mean, causing the DV to re-cross the decision bound (*b*), subsequently cross a change-of-mind bound (*c*), and finally cross the originally correct decision bound, –*θ* (*d*). The grey shaded area indicates a period of uncertainty, or conflict, between the re-crossing of the *θ* bound (*b*) and later crossing of the –*θ* bound (*d*).

All subsequent models of error detection have adopted Rabbitt's broad framework, with debate focusing instead on the precise mechanism by which post-decision processing leads to error detection. Figure 2 illustrates some key model variants. Within a standard DDM framework, errors could be detected as successive crossings of decision boundaries for the two competing responses [31,32] or as ‘double crossings’ of a single decision bound [33]—both close relatives of Resulaj *et al.*'s notion of a change-of-mind bound. Errors can also be detected in terms of the occurrence of uncertainty—or *conflict*—in the decision process after an initial response [34], or as inconsistency between the outcomes of parallel decision processes at different processing stages (e.g. perceptual categorization and response selection) [35]. While varying in their details and precise predictions [32,34], common to all proposals is the claim that metacognitive accuracy judgements depend on post-decision processing.

In effect, applying the framework provided by the DDM and its variants, one is obliged to assume that error detection *reduces to* error correction: that errors are detected whenever corrective activity reaches a criteria level—the change-of-mind bound. However, this conclusion is difficult to reconcile with evidence that error correction and detection are at least partly dissociable, a finding that was again presciently reported in Rabbitt's seminal work. Rabbitt's studies demonstrated that error corrections are a relatively automatic and unreflective consequence of post-decision processing. Thus, they can be extremely fast, occurring within 10–20 ms of the initial error [36], and may be produced even when subjects are instructed to avoid doing so [37]. In contrast, explicit detection and signalling of errors is much slower, voluntary, more prone to interference by distracting tasks, and more sensitive to cognitive decline in normal ageing [29]. Indeed, people sometimes remain unaware of errors that they nevertheless correct [38]. Collectively, these differences suggest that explicit error detection cannot be a mere consequence of post-decision correction: further processing or evaluation must intervene between initial correction and later explicit awareness that an error has occurred. Consistent with this analysis, recent investigations have identified dissociable neural correlates of error correction and detection.

### (b) Neural substrates of error monitoring

Research interest in error monitoring increased substantially following the discovery of scalp EEG potentials that reliably occur time-locked to decision errors. Most studies have used the Eriksen flanker task, in which subjects perform a speeded categorization of a central target (e.g. *H* or *S*), while ignoring flanking distractors that are sometimes compatible (e.g. *HHH*) and sometimes incompatible (e.g. *SHS*) with that target. With modest speed pressure, error rates on incompatible trials can exceed 20 per cent. Following such errors, a negative event-related potential over frontocentral sites is observed within 100 ms of the incorrect response, followed by a later positive wave peaking 200–400 ms later over parietal cortex [31]. Labelled the error-related negativity (ERN/Ne) and error positivity (Pe), respectively, these EEG components have been widely studied to provide insight into error monitoring in healthy and clinical populations.

Converging evidence has identified anterior cingulate cortex (ACC) as the source of the ERN. For example, in simultaneous EEG–fMRI recordings, single-trial ERN amplitude correlates reliably only with activity in a focused ACC source [39]. The source of the Pe is less well characterized, but evidence that it is a variant of the well-studied P3 component [40] would imply widely distributed neural generators in parietal and prefrontal cortex [41]. The P3 has been suggested to reflect the distributed action of norepinephrine released by the locus coeruleus brainstem nucleus in response to motivationally salient events during decision-making [42].

The functional significance of the ERN and Pe is a matter of ongoing debate. Competing theories of the ERN propose a role in error detection, reinforcement learning and conflict monitoring; while theories of the Pe include conscious awareness of errors, affective responses and behavioural adjustments to avoid further errors [43]. These debates notwithstanding, it is now clear that the ERN and Pe dissociably map onto processes related to error correction and error detection, respectively. Thus, ERN onset coincides with the onset of error-correcting activity as revealed through EMG recordings [44], typically around the time of error commission [31], and its amplitude varies with both the speed [45] and probability [46] of error correction. In contrast, Pe amplitude is insensitive to the strength of correcting activity when error detection rates are controlled [47]. Conversely, although both the ERN and Pe are found to covary with subjective ratings of response accuracy [46,48], correlations involving the ERN disappear when the two components are carefully dissociated. In antisaccade tasks in which subjects correct all of their errors but detect only half of them, ERN amplitude is equivalent for aware and unaware errors, whereas the Pe is robustly observed only when subjects detect their errors [38]. Taken together, these findings suggest that whereas the ERN directly indexes automatic post-decision processes leading to rapid error correction, the later Pe is selectively associated with explicit detection and signalling of errors. These results thus provide converging evidence for the view that error correction and detection reflect distinct processes.

A recent study has shed light on specifically how the Pe relates to error detection [49]. Subjects performed a difficult brightness discrimination under speed pressure to induce a mixture of errors resulting from perceptual ambiguity and decision urgency. After each response, they made a binary correct/error judgement, with monetary incentives varied across blocks to encourage either liberal or conservative error signalling. Signal detection analysis indicated that subjects’ accuracy judgements could be well fit by assuming that these judgements reflected a continuum of confidence (from sure correct to sure incorrect), with subjects applying a criterion—a metacognitive *θ*—that varied according to the incentive regime. Critically, error signalling performance was closely related to between-condition and trial-by-trial variation in Pe amplitude (but not the ERN). That is, Pe amplitude appeared to provide a direct neural index of continuously varying decision confidence on which subjects based their metacognitive judgements, with categorical signalling of errors occurring when confidence that the response was wrong exceeded some criterial level.

### (c) Impact of error monitoring on behaviour

The research described above documents the mechanistic and neural basis of error monitoring. A parallel line of research has considered the impact of error monitoring on future behaviour. Much of this work has focused on the finding that subjects usually respond more slowly on trials immediately following errors [27]. Although this effect at least partly reflects the distracting occurrence of a rare event [50], as errors typically are, a consensus view holds that post-error slowing reflects strategic adaptation to prevent further errors [51]. EEG studies have subsequently shown that the degree of observed slowing scales with the magnitude of error-related ERN/Pe activity [46]. Computational models implementing error-related control over distance-to-bound, |*θ|*, account for detailed properties of empirically observed post-error slowing. In one class of model, detection of response uncertainty (conflict) immediately following error commission leads to an increase in the bound—and, hence, more cautious responding—on subsequent trials [52]. Recent extensions of this idea suggest that conflict detection may also be used to adjust the bound dynamically even as a decision is being made [51,53].

Error signals not only support subtle adjustments that optimize online decision-making, they also play a key role in longer term adjustments during learning. Holroyd & Coles [35], for example, suggest that the ERN reflects reinforcement learning of action values. They showed that the ERN migrates in time as new stimulus–response mappings are learned, from initially being triggered by environmental feedback to later being driven by internal representations of the learned mappings, a pattern that mimics the migration of dopaminergic reward prediction error signals from unconditioned rewards to predictive stimuli during conditioning [54]. Meanwhile, fMRI activity in ACC and neighbouring cortex at the time of an incorrect response have been shown to predict response accuracy on later presentations of the relevant stimulus [55,56].

Most studies of post-error adjustments have focused on the ERN and ACC, reflecting wide interest in the role of ACC in reinforcement learning [35,57], rather than on the later Pe component. However, the ERN and Pe typically covary across conditions and, when the two components are dissociated, post-error adjustments are only observed following detected errors on which a Pe component is present [38], suggesting that the latter may be a more direct correlate of the learning mechanisms by which future behaviour is adapted following an error.

## 4. Integrative models of decision confidence and error monitoring

The discussion above highlights convergent evolution in work on decision confidence and error monitoring, suggesting that common principles govern metacognitive judgements regardless of whether errors arise because of intrinsic task difficulty (low drift rate, *δ*) or because of externally imposed decision urgency (low threshold, *θ*). In what follows, we consider the implications of this convergence for future research, both positive (in terms of mutually informative lessons) and negative (in terms of shared limitations).

### (a) Converging themes

Theories of decision confidence and error monitoring each emphasize the importance of post-decision processing, and likewise point to a key dissociation between, on the one hand, continued processing of stimulus information within the decision-making system (leading to changes of mind and error corrections) and, on the other, the formulation and expression of explicit judgements of confidence and accuracy. The literatures have developed these ideas in complementary directions, such that each literature offers valuable lessons for the other. First, whereas confidence is typically characterized as varying along a continuum, and formalized as such in accounts such as the 2DSD model [15], error detection is often characterized as an all-or-none process [32,33]. Thus, according to many current theories of error monitoring, binary yes/no error judgements are an intrinsic feature of the monitoring system rather than a reflection of the arbitrary metacognitive decision that subjects are asked to make. As such, these theories cannot explain how subjects are able to express graded confidence in their accuracy judgements [48,49].

Conversely, the preceding review of work on error monitoring identifies important limitations in current theories of decision confidence. This review suggests that the current debate between decisional locus and post-decisional models of decision confidence will very likely be resolved in favour of the latter, but that even these post-decisional theories will need modification to accommodate evidence that metacognitive awareness (cf. error detection and the Pe) cannot be reduced simply to post-decision processing (cf. error correction and the ERN)—the two are at least partly dissociable. Perhaps more informative still will be exploration of the role of confidence judgements in guiding future actions: whereas research on decision confidence has largely focused on how confidence estimates are derived, a major focus of error-monitoring research has been on how this kind of metacognitive information might be used to modify behaviour both in the short- [27,52,53] and long-term [35,55,56]. Borrowing these insights, we might predict that parametric estimates of confidence could support finer-grained control of behaviour than can be achieved through binary categorization of responses as correct or incorrect—for example, by allowing parametric variation in post-error slowing or by providing a scalar prediction error signal to support reinforcement learning. Thus, confidence estimates could provide useful information in optimizing the rate of learning: one might predict that people will pay greater attention to environmental feedback following responses in which they have less confidence.

### (b) Shared limitations

As well as sharing complementary strengths, the theories also share common weaknesses. In particular, like the models of decision-making on which they are based, current theories of confidence and error monitoring have focused almost exclusively on decisions that are discrete and punctate in time: a decision is made when a boundary is reached [3]; errors are detected when a second change-of-mind bound is crossed [26]; and confidence is estimated at the time of a later metacognitive probe [15]. Characterizing behaviour as a series of discrete decisions, each subject to independent metacognitive scrutiny, is a useful convenience when developing experimental tests and formal models. However, it remains unclear whether the findings will scale up to explain real-world decisions and actions that are fluid, temporally extended and embedded in the broader context of evolving behavioural goals.

Let us revisit for a moment our opening example of cycling along on a winding country lane. Obviously, we do make occasional discrete categorical decisions—for example, when choosing between two available paths at a fork in the road. However, most of our decisions and actions unfold gradually, shaped by our interactions with the environment and an ever-changing stream of incoming sensory information, as exemplified by the continuous, subtle adjustments of handlebars and brakes needed to maintain direction and balance when cycling. Current theories of metacognition require a clear division in time between cognitive decision-making and meta-cognitive evaluation, with the latter beginning when the former ends (‘*post-decision* processing’). However, for continuous and extended actions, there is no definable time-point at which a specific decision is finalized and metacognitive evaluation begins. Most current theories are therefore ill-suited to describing the temporally extended actions, comprising hundreds or thousands of micro-decisions, that characterize everyday behaviour.

Indeed, decisions that initially appear discrete and categorical may turn out, on closer inspection, to be graded and transitory. For example, in the Eriksen flanker task, overt responses occur tightly time-locked to the point at which lateralized activity in motor cortex exceeds a threshold value [58], just as saccadic eye movements appear to be triggered according to a threshold firing rate for LIP neurons [1], suggestive of a fixed decision point after which an action is initiated. However, fine-grained analyses reveal graded and continuous information flow at every stage, even downstream of motor cortex. Thus, within a single trial, motor cortex activity may lateralize first towards one response then towards the other; small EMG twitches in one finger may be followed by full movements of another; and overt actions themselves may vary in force in a graded manner, for example, with incorrect actions executed less forcefully than correct ones [44,58,59]. Meanwhile, categorical or economic judgements about visual information are often preceded by exploratory eye movements, which may themselves constitute interim decisions *en route* to the eventual choice [60]. In such systems, there is no single, final decision point that could mark the beginning of metacognitive evaluation. Detailed analyses of neural markers of metacognition point towards a similar conclusion: error-related EEG activity is not only observed following overt incorrect actions, but also following ‘partial’ errors in which the incorrect muscles twitch, but the incorrect action is not produced [61] and, crucially, in a graded fashion as a function of the level of sub-threshold cortical activity favouring an incorrect response [34]. Metacognition appears to be graded and continuous in just the same manner as the underlying decision process.

Human decision-making also has a continuous quality when viewed over longer timescales, with individual decisions chained into sequences that serve longer-term behavioural goals. Thus, actions that reflect definitive choices at a lower goal level (e.g. movements of the arm, shifts of posture) may constitute reversible, interim choices at a higher goal level (e.g. turning left at a fork in the road), and even a commitment to an individual right or left turn might be just a partial commitment to a yet higher level goal (e.g. reaching a specified destination) [62]. This form of hierarchical structure is built into many recent theories of the computational and neural basis of action selection [63], in which common principles of selection and control are held to operate at each level of hierarchical abstraction [64]. Recent findings indicate that metacognitive processes are similarly sensitive to this hierarchical structure [65]. For example, errors that are equally discrepant in terms of low-level actions are treated very differently according to their impact on global task performance [66]. Little is currently known about the mechanisms by which metacognitive judgements might be embedded in ongoing higher level behaviour in this way.

Thus, a crucial limitation of current metacognitive theories is that they do not reflect the way that our confidence in our actions contributes to fluid, structured sensorimotor behaviour. Rather, they consider that errors are processed in an all-or-nothing fashion, for example, when a post-decision process crosses a metacognitive decision bound. In what follows, we consider another way of thinking about decision confidence that is not subject to these limitations.

## 5. Future directions

Addressing the challenges outlined above will require the development of new models that consider not just discrete, binary choices, but also the kinds of extended, goal-oriented actions that characterize everyday human behaviour. We conclude by considering one promising extension of current decision models, which addresses the issue of the *reliability* of evidence in a manner that opens intriguing new avenues for understanding how metacognitive evaluation might occur for continuous, extended actions.

### (a) Evidence reliability

Formal accounts of categorical decisions, such as the DDM, are often illustrated by analogy to court of law, in which the jury weighs up evidence favouring the innocence or guilt of the defendant [1]. However, this analogy also highlights an inconsistency between current decision models and choices made in the real world: that in the latter, we usually consider the extent to which we *trust* the evidence relevant for a decision. For example, in a law court, evidence from a trustworthy source (for example, official telephone records) might weigh more heavily in a jury's deliberations than evidence from an unreliable source (for example, a witness with a vested interest). Correspondingly, careful signal detection analyses have revealed that human observers are exquisitely sensitivity to evidence reliability when sampling from multiple sources of information [67].

Yet, most currently popular models of perceptual decisions offer no way of expressing the trust or distrust associated with the evidence accumulated; all sources of evidence are combined in the common currency of the DV, which then gives the strength of evidence as a simple scalar value (the vertical location of the diffusing particle in the DDM, or the magnitude of the evidence variable in SDT). Once factored into the DV in this way, the reliability of evidence has no further impact on the decision-making process. This feature contrasts with some accounts of economic choices, in which sensitivity to risk (i.e. outcome variance) as well as value (i.e. outcome mean) has been documented at the behavioural and neurophysiological levels [68,69].

**Figure 3.**
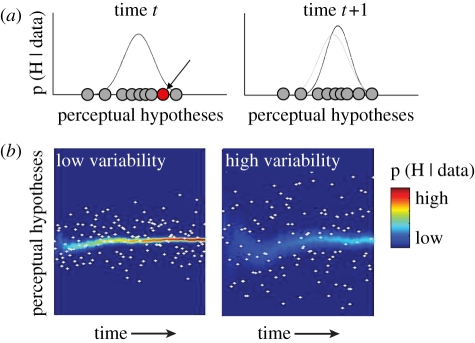
Schematic of a model in which both the mean and variance of information in an array are estimated in a serial sampling framework. (*a*) The left panel shows the posterior probability distribution p (H | data) over a continuous space of possible perceptual hypotheses (e.g. these dots are moving to the right with 30% coherence; this signal is 40% visible; etc.) at a given time, *t*. This distribution reflects the evidence sampled from the stimulus thus far, i.e. between onset and time *t* (grey dots). The new sample received at time *t* is shown in red. Right panel: at time *t* + 1, this distribution (light grey) is updated in the light of the newly sampled information, giving rise to a new probability distribution. In this model, confidence is reflected in the precision of the posterior distribution, i.e. the reciprocal of its standard deviation. (*b*) The evolving posterior probability distribution over perceptual hypotheses (*y*-axis) for each successive time point (*x*-axis; blue–red colourmap; red values indicate higher probabilities). The posterior distribution is updated following the arrival of successive samples with low variance (left panel) or high variance (right panel). Precision of the probabilistic representation of evidence strength increases more rapidly for the low-variability samples.

Mathematically, the reliability of perceptual evidence is orthogonal to its strength, because the mean and the variance represent different moments of a probability distribution. Bayesian models that exploit this point—representing evidence as a probability distribution with a given mean (evidence strength) and variance (evidence reliability)—have been used to calculate ideal estimates of expected value in economic choice tasks [70]. Applied to perceptual decision tasks, the notion would be that the diffusing particle of the DDM is more accurately conceived of as a probability distribution that evolves across samples (figure 3), with the central tendency of that distribution analogous to the vertical location of the particle. Crucially, the variance of this distribution provides additional information—specifically, a representation of evidence reliability in terms of the *precision* of the mean—that is not made explicit in the standard DDM (by *precision*, we mean the inverse of the standard deviation).

Recently, it has been shown that neural network models in which LIP neurons encode the full posterior probability distribution associated with a stimulus can capture behaviour and neural dynamics occurring during psychophysical discrimination tasks in primates [71]. This result suggests that precision may be encoded in the variance of firing rates across a neural population, thus signalling evidence reliability in much the same way that mean firing rate of the population encodes evidence strength [72]. Relatedly, Ratcliff & Starns [73] have recently proposed a model of decision confidence in which the decision-relevant evidence on a single trial is not a scalar estimate, but a distribution of possible values, allowing parametric estimates of confidence in the choice to be calculated as the integral of this distribution falling within graded confidence bounds. Together, these findings open promising new avenues for research into the neural basis of categorical choice.

### (b) Confidence revisited

We propose that evidence strength and reliability are encoded in parallel during evidence accumulation, and that this framework provides an intriguing new way of thinking about decision confidence—as the precision of evidence accumulated during a single trial. This representation of confidence differs in two critical respects from the models described above: it is based on an intrinsic feature of the decision process—variance in the evidence encountered—and it is available continuously and instantaneously. In contrast, existing models suggest that metacognitive evaluations are derived indirectly by comparing representations of the DV from two (or more) discrete time points. Precision thus provides a more suitable basis for metacognitive evaluation in the kinds of temporally extended tasks discussed above, in which no discrete decision point divides cognitive decisions from metacognitive evaluation.

This hypothesis, though speculative at this point, has several attractive features. Firstly, it is consistent with the evidence described above on post-decision processing because instantaneous precision will tend to be highly correlated with later variability in the DV (the basis of most current models of metacognitive judgements): variable evidence will give rise to frequent errors, and these errors will be accompanied by low estimates of precision at the time of the initial response and a high probability of later changes of mind or error correction. Thus, the precision account agrees that existing theories fit the data well, but suggests that they may do so for the wrong reasons. Secondly, the model is able to describe situations in which evidence quality varies even within a single trial [71], something that standard models cannot achieve. In fact, by keeping track of the likely variability of information in the external world, Bayesian accounts can optimally distinguish true state changes in the generative information giving rise to the senses from noise [74]. Precision estimates are thus particularly useful in situations where the causes of perceptual evidence may change unpredictably over time, and as such may provide a better account of the sort of fluid, ongoing sensorimotor integration that characterizes everyday activities such as riding a bicycle.

The hypothesis also leads to clear and testable predictions about the sensitivity of human decision-making to evidence reliability. Firstly, it predicts that variability of evidence within a trial should both reduce accuracy and lengthen RT, because during sequential sampling, the precision of the mean increases more slowly when the samples are drawn from a more variable distribution. This prediction has recently been confirmed in an experiment in which observers made discrimination judgements on the average feature (e.g. colour) of an array of multiple elements presented simultaneously on the screen [75]. Critically, this multi-element averaging task allowed the experimenters to manipulate the mean and the variance of the relevant feature in an orthogonal fashion. The results showed that observers were slower to discriminate more variable arrays, a result that is predicted by the precision account but not by standard accumulation models such as the DDM. Moreover, observers in this study tended to downweight outlying or otherwise untrustworthy evidence, much like a statistician might exclude an outlier from a sample of data. Although this study did not assess subjects’ second-order confidence in their decision, the precision account makes the clear prediction that in the multi-element task, both subjective confidence and the rate of occurrence of changes of mind will depend on array variability to a greater extent than on its mean, a prediction ripe for future testing.

Finally, this conception of decision confidence makes direct contact with broader theories of the role of metacognitive evaluation in behavioural control. In particular, because precision is closely related to the concept of decision conflict [52]—greater variance in evidence should result in greater conflict between competing response options—the theory can inherit ideas from research on decision conflict about how precision estimates might be used to guide both current performance (e.g. through dynamic modulation of decision bounds) [51,53] and future behaviour (e.g. through modulation of learning rate in relation to environmental signals of success or failure) [70]. As such, the precision model not only provides a formally specified account of decision confidence, but also leads to immediate suggestions about the use of confidence judgements in the optimization of behaviour.

## 6. Conclusion

Formal models such as the DDM have proved extremely valuable in understanding human and animal decision-making, by situating experimental observations of behaviour and neural activity within a precisely specified and normatively motivated framework. Direct extensions of these models have proved equally useful in probing the metacognitive processes by which we evaluate and express our degree of confidence in our decisions. In particular, significant convergence in methods and theories of decision confidence and error monitoring suggest that common principles may govern different types of metacognitive judgements.

There is nonetheless important scope for current models to consider decision-making and metacognitive evaluation in situations that encompass not only simple, punctate choices, but also the kinds of extended, goal-directed decisions and actions that typify human behaviour outside the experimental laboratory. We have proposed one such extension: the hypothesis that people are sensitive not only to the strength of evidence they encounter as they make a decision, but also to the reliability of that evidence. This simple proposal has far-reaching implications: it immediately suggests a novel source of information—evidence precision—that could guide metacognitive evaluation. Future developments in theories of human decision-making promise to have similarly profound implications for our understanding of the way in which people evaluate their decisions in the service of adapting and optimizing those decisions in the face of an uncertain, complex and ever-changing environment.
